# “What Is a Step?” Differences in How a Step Is Detected among Three Popular Activity Monitors That Have Impacted Physical Activity Research

**DOI:** 10.3390/s18041206

**Published:** 2018-04-15

**Authors:** Dinesh John, Alvin Morton, Diego Arguello, Kate Lyden, David Bassett

**Affiliations:** 1 Department of Health Sciences, Northeastern University, Boston, MA 02115, USA; arguello.d@husky.neu.edu; 2 Department of Kinesiology, Recreation, and Sport Studies, University of Tennessee, Knoxville, TN 37996 USA; amorto16@vols.utk.edu (A.M.); dbassett@utk.edu (D.B.); 3 KAL Research/Consulting, Denver, CO 80206, USA; katelyden6@gmail.com

**Keywords:** Step-detection, ActiGraph, Pedometer, acceleration, physical activity

## Abstract

(1) Background: This study compared manually-counted treadmill walking steps from the hip-worn DigiwalkerSW200 and OmronHJ720ITC, and hip and wrist-worn ActiGraph GT3X+ and GT9X; determined brand-specific acceleration amplitude (g) and/or frequency (Hz) step-detection thresholds; and quantified key features of the acceleration signal during walking. (2) Methods: Twenty participants (Age: 26.7 ± 4.9 years) performed treadmill walking between 0.89-to-1.79 m/s (2–4 mph) while wearing a hip-worn DigiwalkerSW200, OmronHJ720ITC, GT3X+ and GT9X, and a wrist-worn GT3X+ and GT9X. A DigiwalkerSW200 and OmronHJ720ITC underwent shaker testing to determine device-specific frequency and amplitude step-detection thresholds. Simulated signal testing was used to determine thresholds for the ActiGraph step algorithm. Steps during human testing were compared using bias and confidence intervals. (3) Results: The OmronHJ720ITC was most accurate during treadmill walking. Hip and wrist-worn ActiGraph outputs were significantly different from the criterion. The DigiwalkerSW200 records steps for movements with a total acceleration of ≥1.21 g. The OmronHJ720ITC detects a step when movement has an acceleration ≥0.10 g with a dominant frequency of ≥1 Hz. The step-threshold for the ActiLife algorithm is variable based on signal frequency. Acceleration signals at the hip and wrist have distinctive patterns during treadmill walking. (4) Conclusions: Three common research-grade physical activity monitors employ different step-detection strategies, which causes variability in step output.

## 1. Introduction

Wearable activity monitors are commonly used to detect steps to quantify physical activity (PA) [1]. While this seemingly standard metric should enable a direct comparison of PA among studies using step-counters, validation studies report discrepancies in steps from different commercial activity monitors [2,3,4,5]. This may be attributable to variability in criteria used by the devices to translate human movement into steps.

Furthermore, there may be within-brand differences in step-output. For example, steps using ActiGraph’s ActiLife software on data from newer devices (i.e., model GT1M and higher), yield step-outputs that are not comparable to the older 7164 [6,7] and location of wear (hip vs. wrist) impacts ActiGraph step-output [8]. A substantial proportion of current knowledge on associations between objectively measured PA and health results from the nationally representative 2003–04/2005-06 National Health and Nutrition Examination Survey (NHANES), which used the ActiGraph 7164 [9,10,11]. However, the latest cycle of the NHANES (2011–12/2013-14) used a wrist-worn GT3X+ [12]. Additionally, while steps computed using ActiLife is most common in PA research, real-time step output is also available to a user via the device display screen in the new GT9X. However, the latter is computed using a real-time step-counting algorithm that is different from that in ActiLife. The real-time algorithm on-board the GT9X uses tri-axial (as opposed to uni-axial acceleration data in ActiLife), and filters out steps that are accumulated in bouts lasting less than 2 and 10 s on the hip and wrist, respectively. Thus, in the context of objectively measuring PA using motion sensors, the answer to the question “what is a step?” may be contingent on device-specific *proprietary* strategies used to detect and translate movement acceleration into steps.

Thus, our study examined the abovementioned question by first investigating step-output comparability among three devices that have significantly impacted PA research. This was followed-up with a determination of device-specific parameters used to define and detect a step. The three devices were the mechanical Yamax Digiwalker SW200, the piezoelectric cantilever accelerometer-based Omron HJ720ITC, and MEMs capacitive sensor-based ActiGraph monitors. A reason for selecting these three devices was that unlike various consumer-based devices that are currently available, these three brands of devices have significantly influenced the creation of commonly used step goals (e.g., 10,000 steps/day) [13,14,15] and/or have been widely used in both population-level studies (e.g., NHANES) [16,17,18,19,20] and in clinical trials [15,21,22,23]. However, these devices also use unknown proprietary strategies to define a step. Differences in proprietary strategies that dictate how a step is detected, may result in the invalidation of a step as a universal metric of PA and compromise inter-study comparisons of this metric. To our knowledge, no study has systematically determined device-specific step-detection parameters among these three brands of activity monitors. Such an examination will provide an improved understanding of “why” movements recorded as a step by one device are not classified as such by another device. 

Thus, Aim 1 of our study compared manually-counted steps during treadmill walking to those from the hip-worn Digiwalker SW200 and Omron HJ720ITC, and steps from hip and wrist-worn GT3X+ and GT9X monitors processed using ActiLife software. Aim 2 determined device-specific raw acceleration amplitude (g-value) and/or frequency (Hz) thresholds to record steps. Results of Aim 2 may explain likely differences in step outputs among devices in Aim 1. A secondary aim of our study was to quantify features of high-resolution acceleration signals detected at the hip and the wrist and identify/describe visually distinctive patterns in the signals during walking. Such findings may further explain variability in output from Aim 1 and inform the development of superior non-proprietary step-detection methods when using accelerometer-based step-counters.

## 2. Materials and Methods

**Figure 1:**
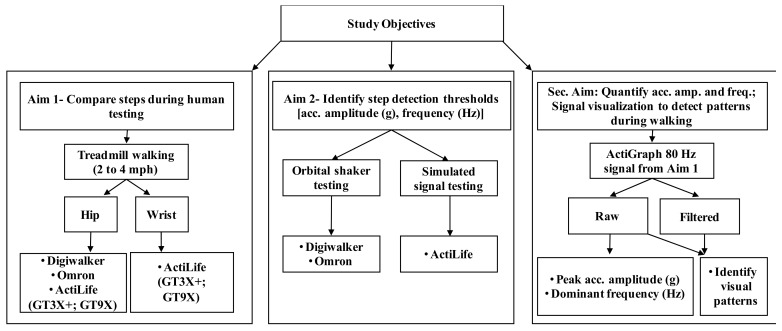
Conceptual outline of study aims and associated experimental protocols (acc. = acceleration; amp. = amplitude; freq.= frequency).

Twenty participants (Age: 26.7 ± 4.9 years; BMI: 26.1 ± 3.5 kg/m^2^; 12 males and 8 females; all right-handed) with no gait abnormalities and who were experienced in using a treadmill volunteered for this study. The study was approved by the Northeastern University Institutional Review Board and all participants provided written informed consent prior to commencing study participation. Figure 1 is a conceptual outline of study aims and associated experimental protocols, which are detailed below (Figure 1).

### 2.1. Devices

**Figure 2:**
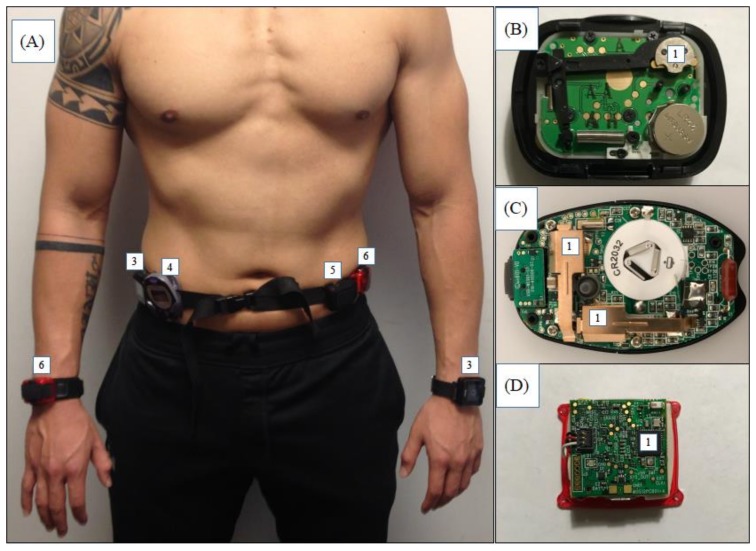
Participant with the ActiGraph GT3X+ (#6), GT9X (#3), the Omron HJ720ITC (#4), and the Digiwalker SW300 (#5). Figures (**B**–**D**) depict the sensing elements (#1) inside the three devices.

The hip-worn Digiwalker SW200 is a coiled spring-suspended pendulum-based mechanical device, which counts steps when vertical acceleration during hip movement sufficiently displaces the pendulum to complete an electrical circuit (Figure 2). The Omron HJ720ITC is typically worn on the hip, has two piezoelectric sensors that are placed perpendicular to each other on its circuit board, and uses proprietary criteria to detect steps in real-time. The ActiGraph GT3X+ and GT9X can be worn on both the hip and wrist. Data from these capacitive accelerometer-based devices [24] can be post-processed to obtain steps using ActiLife software (ActiGraph LLC., Pensacola, FL, USA).

### 2.2. Aim 1—Human Treadmill Testing

Participants wore a single Digiwalker SW200, Omron HJ720ITC, GT3X+, and GT9X at the waist using a snug elastic belt (Figure 2). The GT3X+ and the GT9X were positioned in line with the anterior axillary line on the left and right hip. The Omron HJ720ITC and Digiwalker SW200 were placed medially adjacent to the ActiGraph monitors. A GT3X+ and GT9X were worn at the most distal location on the left and right wrist. Monitor locations (left and right) were counterbalanced among participants to account for any confounding due to placement. Device placement was based on typical procedures followed in PA research, i.e., as per the manufacturer’s recommendation (Figure 2).

The experimental protocol consisted of treadmill walking at 11 speeds ranging between 0.89 and 1.79 m/s (2 to 4 mph) in increments of 0.089 m/s (0.2 mph). The protocol yielded steady-state step-counts for one minute at each speed. A researcher manually counted steps (criterion) during the protocol. A researcher verified each treadmill speed using a handheld tachometer (Shimpo DT-105A, Nidec-Shimpo America Corp., Itasca, IL., USA).

### 2.3. Aim 2—Testing to Examine Step-Detection Thresholds for Acceleration Amplitude (g) and Frequency (Hz)

Processing simulated signals of known frequency and amplitude using device-specific algorithms lends maximum control and accuracy in determining the minimum acceleration required to detect a step (i.e., step-counting threshold). However, the Omron HJ720ITC and the Digiwalker SW200 do not have associated software or software features that facilitate such processing. Hence, to determine step-detection thresholds, we conducted shaker-testing for the Omron HJ720ITC and Digiwalker SW200, and simulated sinusoidal signal testing for the ActiGraph algorithm.

#### 2.3.1. Shaker Testing (Omron HJ720ITC and Digiwalker SW200)

Our orbital shaker (Model 1231A89, Thomson Scientific, London, UK) maintains a monitor in a fixed spatial orientation, while subjecting the device to a circular motion in the horizontal plane at adjustable frequencies and radii ranging from 0.25 to 8.0 Hz (15 to 480 rpm) and 1.6 to 16 cm, respectively. Both devices were tested at 4 different radii: 2.1, 2.6, 4.5, and 12.0 cm. Since, acceleration thresholds required to commence step counting may be different for these devices, an initial trial was conducted to determine monitor-specific testing frequencies. Testing frequencies were increased at the rate of 0.016 Hz (1 rpm). Each monitor then underwent 10 trials that yielded a minute of steady-state shaker testing at each testing frequency. The Omron HJ720ITC was tested from 0.97 to 1.17 Hz (58 to 70 rpm) at each of the testing radii. The Digiwalker SW200 was tested from 3.73 to 3.83 Hz (224 to 230 rpm) at 2.1 cm, from 3.35 to 3.45 Hz (201 to 207 rpm) at 2.6 cm, from 2.53 to 2.63 Hz (152 to 158 rpm) at 4.5 cm, and from 1.50 to 1.63 Hz (90 to 98 rpm) at 12 cm.

Devices were fastened to the shaker plate such that there was no movement artifact due to placement and each brand of monitor was tested separately. The protocol was video-recorded using a stationary high-speed camera placed at a height of approximately 30-cm from the shaker plate. Time-stamped video recordings were immediately used to retrieve and record steps/min at each frequency from the display screens of the two devices.

#### 2.3.2. Simulated Sinusoidal Signal Testing for ActiGraph

Simulated signal testing has been used to test characteristics of ActiLife software [25,26,27]. Signals were simulated at a sampling rate of 80 Hz for frequencies ranging from 0.2 to 2.4 Hz, in increments of 0.2 Hz. For each frequency, signals were generated at amplitudes ranging between 0.01 to 0.2 g in increments of 0.01 g. Simulated signals were generated using MATLAB (R2016B, MathWorks, Natick, MA., USA) and were processed using ActiLife v6.13.3 to generate step-output [25,26].

### 2.4. Secondary Aim—Quantifying Selected Signal Features and Analysis of Acceleration Waveforms (Raw/Filtered) during Treadmill Walking

We quantified specific amplitude and frequency characteristics of the high-resolution acceleration signal from a GT3X+ worn at the hip and wrist. Walking is rhythmic and produces a relatively consistent waveform pattern with acceleration peaks during a step. Average peak acceleration amplitudes were derived from steady-state walking steps at each treadmill speed. Frequency characteristics included the dominant and 2nd dominant signal frequencies derived using Fourier Transform in MATLAB. We also report visually detectable time-domain patterns in the acceleration signal during walking for raw and filtered acceleration (0.25–2.5 Hz). This bandwidth was selected for this demonstration as previous work has shown that it is sufficient to capture movement related to human ambulation [28].

### 2.5. Data Analyses

Step outputs during human, shaker, and simulated signal testing were compiled into a Microsoft Excel database. All ActiGraph data (80 Hz sampling rate) were processed using ActiLife v6.13.3.

For the treadmill protocol (aim 1), mean steps and estimation bias (95% confidence intervals) were derived using IBM^®^ SPSS^®^ Statistics software (*v.* 23, Armonk, NY, USA). Bias was defined as steps from direct observation minus monitor output. Acceleration amplitude and/or frequency thresholds required by a device to detect steps were derived from shaker and sinusoidal signal testing (aim 2). Orbital shaker testing subjected the Digiwalker SW200 and the Omron HJ720ITC to sinusoidal acceleration in the *x* and *y*-axes of the device where each revolution of the orbital shaker generates a curve that contains the two extrema of the acceleration sine wave with a zero-g value crossing. Hence, one revolution is one step. Details on computing peak acceleration amplitude during orbital shaker testing can be found elsewhere [29].

## 3. Results

### 3.1. Aim 1–Human Treadmill Testing

Table 1 depicts mean criterion and estimated steps and estimation bias (95% confidence intervals) from hip and wrist-worn devices during the treadmill protocol. Statistically significant underestimations were found for the hip-worn Omron HJ720ITC at 1.61 m/s (3.6 mph) and 1.79 m/s (4.0 mph), the hip-worn Digiwalker SW200 at 0.89 m/s (2.0 mph), 0.98 m/s (2.2 mph), and 1.16 m/s (2.6 mph), and both hip and wrist outputs from the GT3X+ and GT9X at all speeds. ActiGraph monitors were least accurate in detecting steps (Table 1).

### 3.2. Aim 2–Testing to Examine Step-Count Thresholds for Acceleration Amplitude (g) and Frequency (Hz)

#### 3.2.1. Digiwalker SW200

Table 2 contains mean steps detected during the shaker testing protocol for the Digiwalker SW200 and the corresponding acceleration amplitude computed at the testing frequencies. Our findings suggest that a total acceleration amplitude of at least 1.21 g is required to sufficiently displace the spring-levered mechanism in the Digiwalker SW200 and count a step. We used the lowest g-force when a step is detected to represent step-detection threshold. This accounts for marginal decrements in the g-force produced (during few to several rotations) that is attributable to friction and other environmental factors that impact the rotation of the shaker plate.

#### 3.2.2. Omron HJ720ITC

The Omron HJ720ITC counts a step when the detected motion satisfies both an acceleration amplitude and frequency threshold. Movement with a minimum acceleration amplitude of 0.10 g is required to detect steps (Table 2). At a radius of 2.1 cm, step counting did not commence until a frequency of 1.10 Hz (66 rpm), which generates an acceleration of 0.10 g. At all other radii, i.e., 2.6, 4.5, and 12 cm, acceleration amplitude at 1 Hz (60 rpm) and above were greater or equal to 0.10 g (range: 0.10 to 0.65 g). Our findings suggest that the Omron HJ720ITC records steps for those peaks in the acceleration waveform that have a minimum amplitude threshold of ±0.10 g, but only when the waveform has a dominant frequency ≥ 1 Hz (Table 2).

#### 3.2.3. ActiGraph’s ActiLife Algorithm

Sinusoidal signal testing demonstrated that the step-detection response of the ActiLife algorithm is variable based on both acceleration amplitude and frequency. Table 3 is a grid that shows the relationship between raw acceleration amplitude and frequency for the ActiLife step counting algorithm. The algorithm was most sensitive between frequencies of 0.7 and 0.8 Hz and required an acceleration amplitude of at least 0.07 g to detect a step (Table 3).

### 3.3. Secondary Aim-Quantifying Selected Signal Features and Visual Analysis of Acceleration Waveforms (Raw/Filtered) during Treadmill Walking

**Figure 3:**
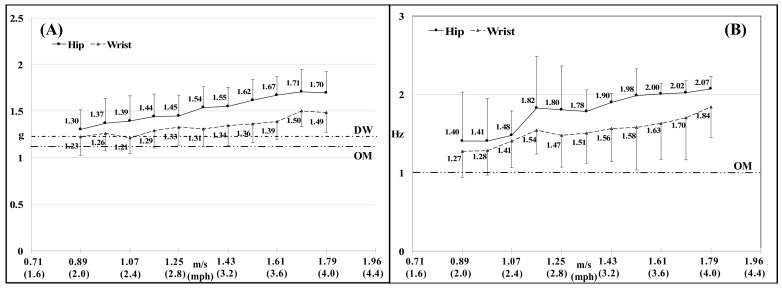
Mean ± SD of peak high-resolution (80 Hz) acceleration detected in the *y*-axis of the GT3X+ at the hip (**A**) and wrist (**B**) during walking at various speeds. Dotted lines in 3A indicate step-detection total acceleration thresholds for the hip-worn Digiwalker SW200 (1.21 g) (DW) and the Omron HJ720 ITC (0.10 g) (OM). Please note that although the piezoelectric Omron HJ720 ITC is not sensitive to the force of gravity, we added the gravity component to denote the threshold for the Omron because, the figure depicts total acceleration (gravity component + movement acceleration). Dotted line in 3B is the frequency threshold for the Omron HJ720 ITC (1 Hz). We do not depict thresholds for ActiLife as it is variable based on signal frequency due to bandpass filtering. Additionally, specifications of the filter are proprietary and hence it is not possible to scale the filtered signal thresholds identified in the study to an unfiltered acceleration (g) value in Figure 3A.

Overall, peak acceleration and the dominant signal frequency at the hip and wrist increased with speed (Figure 3A,B). Supplementary Table S1 contains values for the 2nd dominant frequency of hip and wrist movement (Figure 3).

**Figure 4:**
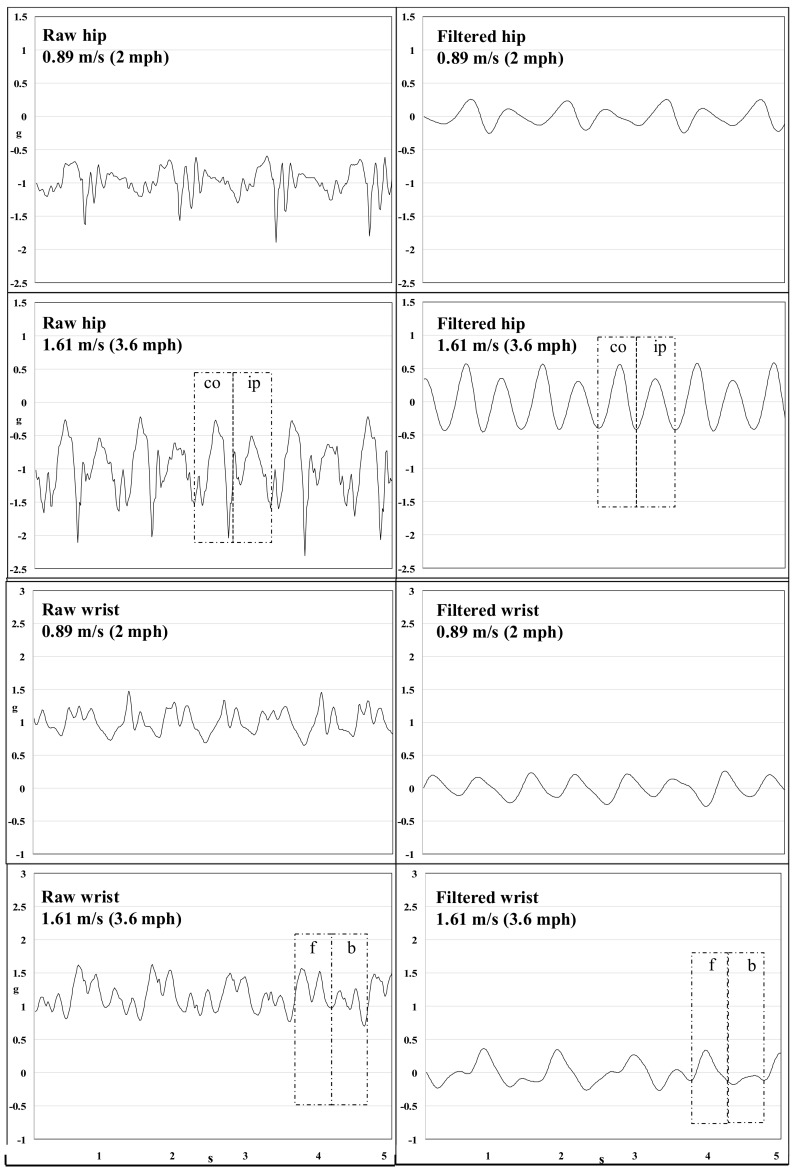
*y*-axis acceleration (5 s; 80 Hz) from a hip and wrist GT9X on a single participant and their corresponding bandpass filtered signals (0.25-2.5 Hz) processed using a fourth-order Butterworth filter. Dotted boxes indicate the same steps in the raw and filtered signals. ‘co’= contralateral step (step taken by the leg on the same side as the hip-worn device); ‘ip’= ipsilateral step (step taken by the leg on the opposite side as the hip-worn device); ‘f’= forward arm swing; ‘b’= backward arm swing.

Signal visualization (*y*-axis and tri-axial vector magnitude) revealed a consistent pattern where the shape of the acceleration waveforms for consecutive steps looked different, but alternate step patterns were similar. Figure 4 demonstrates this pattern using the signal detected in the *y*-axis of a GT9X at the hip and wrist. To further explore this phenomenon, we conducted video analyses of five participants who wore a GT9X at the dominant hip and the non-dominant wrist while walking at 1.61 m/s (3.6 mph) on a treadmill. Time-stamped videos were synchronized with the *y*-axis waveform from the GT9X at each site. For the hip, we defined an ipsilateral step as that by the leg on the same side as the hip on which the device was worn. The contralateral step was that taken by the opposite leg. Ipsilateral steps taken in concert with a forward arm swing of the non-dominant wrist generated acceleration curves that were larger than contralateral steps and the backward arm swing, respectively. Additionally, compared to raw data, filtered data are smoother and resemble sine-waves. The latter may simplify the detection of movements associated with a step. Thus, it is likely that monitor manufacturers may employ some form of acceleration signal filtering when detecting steps (Figure 4).

## 4. Discussion

The overarching goal of this study was to examine how inter-device differences in proprietary step-detection strategies impact the comparability of step outputs during human movement. For this, we used devices that have significantly contributed to existing knowledge on the impact of steps/day on health. We first present a discussion that integrates findings from human testing in our study (Aim 1) and previous work with findings from our investigation of step-detection strategies in the three brands of activity monitors (Aim 2). This is followed by a discussion on observations from the secondary aim.

### 4.1. Aims 1 and 2—Human Treadmill Testing and Examining Step-Count Thresholds for Acceleration Amplitude (g) and Frequency (Hz)

#### 4.1.1. Digiwalker SW200

The device in our study required a total raw g-force threshold of 1.21 g to count a step. We placed the device flat on the shaker table, and subjected it to orbital motion in the horizontal plane. Such a placement eliminates the gravity component (1 g) that is exerted on the spring-based pendulum when the device is worn on the body (vertical plane). Thus, the additional vertical acceleration (beyond gravity) required to count a step is 0.21 g. Our empirically determined threshold of 0.21 g for the Digiwalker SW200 is lower than the previously reported threshold of 0.35 g [30]. Supporting evidence for our estimate can be inferred from the raw acceleration detected by the ActiGraph at the hip (Figure 3A). At speeds between 0.89 m/s (2 mph) and 1.25 m/s (2.8 mph), step-detection accuracy increased from 79 to 92%. At these speeds, mean ± SD of peak acceleration (including the gravity component) ranged between 1.30 ± 0.21 to 1.45 ± 0.22 g. Given that (i) peak acceleration increases with increasing speed; (ii) there is considerable variability in peak acceleration at each speed (~0.21 g, Figure 3A); and (iii) contralateral steps generate lower acceleration than ipsilateral steps (Figure 4); increasing walking speed results in an incremental proportion of steps that satisfy the acceleration threshold to detect a step, thereby resulting in a corresponding increase in step-detection accuracy (Table 1).

#### 4.1.2. Omron HJ720ITC

The acceleration amplitude threshold of 0.10 g in the Omron HJ720ITC does not include the gravity component, which is attributable to the use of piezoelectric sensors to detect steps. Piezoelectric sensors do not detect static acceleration and produce an electric voltage that is proportional to movement only. We found that beyond the 0.10 g threshold, subsequent increases in the amplitude of acceleration did not alter the frequency threshold of 1 Hz (60 rpm). Thus, the hip-worn Omron HJ720ITC may cause larger step-detection errors during slow walking that may be attributable to a low step frequency rather than an insufficient acceleration amplitude because during walking, it is improbable that acceleration at the hip will not exceed a threshold of 0.10 g. Correspondingly, Jehn et al. reported that a step-frequency between 80–90 steps/min was necessary for the Omron HJ720ITC to accurately detect steps [31].

#### 4.1.3. ActiLife

The ActiLife step-counting algorithm is based on proprietary thresholding of the bandpass filtered uni-axial (*y*-axis) acceleration signal. Here, the filtered acceleration waveform needs to cross both positive and negative values of a fixed proprietary acceleration amplitude threshold to qualify as a step. Consecutive positive and negative crossings of the amplitude threshold are equidistantly separated by a zero-g crossing, which is used as an event that marks a valid step [32]. Bandpass filtering eliminates the gravity component, which yields a zero-g crossing and a low amplitude threshold for step-detection (see Figure 4).

*Variability in acceleration thresholds:* Although step-counting in ActiLife is based on a fixed acceleration amplitude threshold, we found that at different signal frequencies, the step-detection amplitude threshold is variable (Table 3). This is due to bandpass filtering of the raw acceleration signal that occurs prior to signal thresholding to detect steps (described above). ActiGraph’s bandpass filter is maximally sensitive at 0.75 Hz. As frequency components in the signal move away from 0.75 Hz within the passband (0.25 to 2.5 Hz), the amplitude of the signal is reduced symmetrically [27]. At 0.29 and 1.66 Hz, signal amplitude is reduced to 70.7% of that at 0.75 Hz [27]. This filter response explains peak sensitivity to detect steps between 0.7 and 0.8 Hz and the need for increasing acceleration amplitude (g-value) with increasing frequency to detect steps (table 3). During commonly preferred walking speeds of approximately 1.34 to 1.56 m/s (3 to 3.5 mph) in normal adults [33,34], the dominant frequencies in the acceleration signal at both the hip and wrist in our study were close to or greater than 1.66 Hz [1.78 ± 0.28 Hz at 1.34 m/s (3 mph) at the hip; 1.63 ± 0.46 Hz at 1.52 m/s (3.4 mph) at the wrist (Figure 3B)]. 

*Effect of monitor location on step*-*counting:* We are unaware of the reasons causing differences in step output between the hip-worn GT3X+ and GT9X. However, we discuss causes for differences between hip and wrist-worn ActiGraph devices and between wrist-worn GT3X+ and GT9X devices.

The ActiLife step-counting algorithm was developed for hip-worn devices and uses acceleration from only the *y*-axis to detect steps. Acceleration detected at the wrist are smaller in magnitude than those at the hip during walking at the same speed (Figure 4). This will result in fewer instances where the acceleration waveform satisfies thresholding criteria, especially at slower walking speeds. However, during a 24-h period under free-living conditions, a wrist-worn GT3X+ resulted in higher step counts than a hip-worn device [8]. This paradoxical finding may be explained by the fact that many activities of daily living involve hand movements that result in the detection of several false-positive steps.

**Figure 5:**
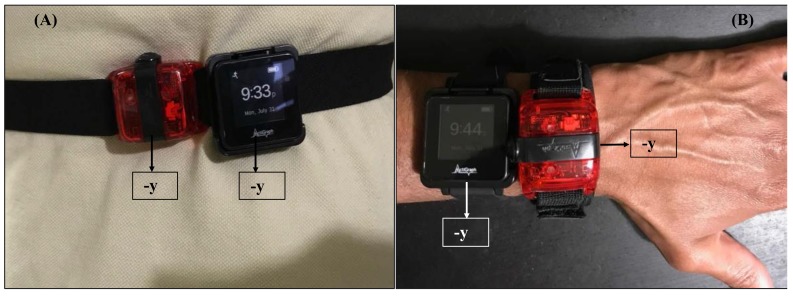
Orientation of the axes in the GT3X+ and GT9X when worn on the hip (**A**) and wrist (**B**), respectively.

A significant source of error between a wrist-worn GT3X+ and GT9X is a discrepancy in the orientation of the tri-axial coordinate system due to a dissimilarity in “form-factor” (Figure 5A,B). This inter-device discrepancy in axis orientations will significantly impact step counting accuracy between the GT9X and other ActiGraph devices at the wrist (Figure 5).

Differences in individual gait, location of wear, and walking speed may impact thresholding of the signal in ActiLife, and thereby step-counting accuracy. These factors may yield a filtered signal that may not (i) cross both negative and positive thresholds required to count a step, or (ii) produce a zero crossing.

### 4.2. Secondary Aim—Quantifying Selected Signal Features and Visual Analysis of Acceleration Waveforms (Raw/Filtered) during Treadmill Walking

Although stepping involves displacement of the body’s center of mass, which results in hip movement, the magnitude of peak-to-peak acceleration between two consecutive steps varies for hip-worn devices. During the contralateral step, i.e., a step taken by the left leg when the device is worn on the right hip, and vice versa, the sensor captures attenuated accelerations (Figure 4). This is because the device is not on the hip that is causing the step. At slower walking speeds, smaller accelerations detected during the contralateral step may not satisfy the step-detection criteria of the Digiwalker SW200 and ActiGraph, which may yield fewer step-counts (Figure 4).

At the wrist, the step waveform during the forward swing of the arm, tends to produce a distinctly larger waveform as compared to that during the backward arm swing. However, during walking, arm movement may not always reflect whole body movement (e.g., when holding an umbrella). Thus, arm swing patterns during walking may be less consistent than those observed at the hip. Signal processing techniques that are not limited to simple thresholding, but which also identify frequency features that are inherent in the signal during walking, may be useful to detect steps when wrist movement is restricted during ambulation.

### 4.3. Strengths and Limitations

A strength of this study is the use of three proprietary devices that have significantly influenced PA research. These devices use different hardware and strategies to detect steps, a universal metric of PA. Our study used human, mechanical, and software-based testing to demonstrate variability in step-detection parameters among tested devices, and how variability translates to discrepancies in steps. Weaknesses include limiting human testing to treadmill walking that may not represent natural over-ground walking, and exclusion of popular consumer wearables.

## 5. Conclusions

The Digiwalker SW200, the Omron HJ720ITC and the ActiGraph devices have significantly influenced the understanding of the dose–response between steps/day and health outcomes. However, the parameters to define and detect a step in all three devices are different.

- The Digiwalker SW200 has a movement acceleration step-detection threshold of 0.21 g.
- The Omron HJ720ITC detects a step when movement acceleration peak is ≥ 0.10 g, but only when the dominant frequency of the signal is ≥ 1 Hz. The ActiLife algorithm primarily relies on thresholding of the band-pass filtered acceleration signal from a single axis. A step is detected only when the signal has a zero-crossing and crosses both a positive and negative threshold on either side of the zero-crossing.
  ○ Step-detection threshold for the ActiGraph’s ActiLife algorithm is variable based on signal frequency due to signal filtering.
  ○ Our findings suggest that current step-detection strategies in ActiLife may be invalid for step detection using devices worn at both the hip and the wrist.

### 5.1. Implications of Variability in Proprietary Step-Detection Parameters

Similar to our study, previous work found that steps from hip-worn ActiGraphs and Digiwalker perform poorly at slower walking speeds [<~1.12 m/s (2.5 mph)], while Omron devices detect most steps at speeds above 0.89 m/s (2 mph) [3,35,36]. Another study reported that the Omron HJ720ITC was unreliable at slow walking speeds <0.83 m/s (1.85 mph.). Thus, the proprietary step detection thresholds in these three devices may be inappropriate to detect steps during slow ambulation, particularly when used in populations where gait speed is markedly reduced (e.g., older adults, disabled populations). Studies comparing the hip-worn Omron HJ720 ITC, the Digiwalker SW200, and ActiGraph devices to a criterion ankle-worn step-counter have reported significant underestimations of steps during 24-h free living [3,37,38]. Additionally, existing evidence that the Omron HJ720ITC does not count steps accumulated in durations lasting less than 4 s [38] in combination with findings on the frequency threshold from our study suggest that the step-counting algorithm in the Omron HJ720ITC was designed to capture purposeful, rhythmic bouts of walking. However, activities of daily living often involve short/intermittent stepping bouts that may last few to several seconds [39,40]. Thus, several of these steps may go undetected by the Omron HJ720ITC.

From a public health perspective, our findings may explain differences in steps/day estimates for the US population [20,41]. One study using a spring-levered pedometer reported that adults averaged approximately 5117 steps/day [41]. Conversely, based on NHANES 2005–2006 accelerometer data, American adults accumulate 9676 steps/day. This nearly 2-fold difference in steps/day from two US population-level studies may be attributable to differences in step-detection parameters in the two devices used in these studies. Given a rapid turnover of new models for each brand of popular consumer wearables, such devices are likely more suitable for end-users in the general population. However, the adoption of consumer wearables in PA and public health research is quickly increasing [42,43]. Most of these devices use proprietary methodologies to detect steps and hence, differences may exist in the types of movements that are captured as steps. For example, one study examining steps during numerous simulated activities of daily living (including ambulation) demonstrated significant differences in step outputs between the Apple watch and ground truth [44]. Another study using a wrist-worn Fitbit reported that the device underestimated free-living steps by up to 30% as compared to directly-observed ground truth [45]. Recent work found that proprietary methods in the Apple Watch and the FitBit Blaze yield significantly different steps during 19 different activities and during free-living conditions [46]. One factor may be that the Apple watch likely uses a summarized acceleration derived from each axis of a triaxial accelerometer as compared to the FitBit Blaze, which uses individual information from each of the three axis [46].

### 5.2. Outlook for the Future

Devices and methodologies that use proprietary and/or rudimentary acceleration threshold-based strategies may insufficiently capture steps during purposeful ambulation and activities with intermittent movements. Furthermore, to establish steps as a standard metric of PA, it may be necessary for the research community to have a consensus on movements (and related acceleration signal characteristics) required to represent a step. Such a consensus framework may then be used to expedite the development of *a non*-*proprietary, open*-*source* step-detection methodology that does not rely solely on simple acceleration thresholds, but considers multiple acceleration features to classify movements as a step. This may help to standardize the step as a metric of PA. Similar approaches may be necessary when considering new methods for clinical populations to ensure uniformity across devices used in a specific pathological condition that alters gait.

## Figures and Tables

**Table 1:** Mean (SD) and bias (95% confidence intervals) for steps from the hip-worn Omron, Digiwalker, GT3X+, and GT9X and for the wrist-worn GT3X+ and GT9X during the treadmill protocol. Bias was computed as criterion minus estimated steps; grey cells indicate a significant over, or underestimation of device output.

Speed [m/s (mph)]	Criterion	Omron		Digiwalker		GT3X+ Hip		GT9X Hip		GT3X+ Wrist		GT9X Wrist	
	Mean (SD)	Mean (SD)	Bias (95% CI)	Mean (SD)	Bias (95% CI)	Mean (SD)	Bias (95% CI)	Mean (SD)	Bias (95% CI)	Mean (SD)	Bias (95% CI)	Mean (SD)	Bias (95% CI)
0.89 (2.0)	98 (13)	93 (31)	5 (−8, 17)	77 (34)	21 (7, 34)	58 (19)	39 (29, 49)	43 (19)	58 (50, 67)	53 (17)	46 (38, 54)	31 (14)	68 (60, 77)
0.98 (2.2)	101 (10)	95 (22)	6 (−5, 17)	85 (29)	15 (4, 27)	72 (19)	28 (17, 38)	42 (22)	63 (52, 74)	68 (15)	38 (29, 47)	37 (18)	66 (57, 74)
1.07 (2.4)	104 (10)	102 (13)	1 (−2, 5)	96 (22)	8 (−1, 16)	85 (18)	18 (9, 27)	45 (21)	62 (53, 72)	74 (17)	38 (27, 48)	40 (15)	66 (58, 73)
1.16 (2.6)	108 (9)	106 (14)	2 (−1, 5)	100 (22)	8 (1, 15)	90 (20)	18 (8, 28)	46 (21)	64 (55, 73)	77 (20)	32 (21, 43)	41 (15)	68 (61, 74)
1.25 (2.8)	112 (9)	112 (11)	1 (−2, 3)	103 (28)	9 (−2, 21)	97 (15)	15 (7, 22)	48 (23)	66 (55, 76)	79 (19)	35 (24, 46)	46 (13)	68 (60, 76)
1.34 (3.0)	115(10)	117 (14)	−3 (−7, 2)	112 (11)	2.4 (1, 5)	104 (10)	11 (6, 15)	53 (25)	64 (53, 75)	82 (24)	39 (26, 53)	50 (12)	67 (61, 74)
1.43 (3.2)	117 (8)	117 (10)	0 (−3, 3)	116 (10)	2 (−1, 3)	109 (8)	8 (5, 11)	56 (27)	63 (51, 75)	85 (23)	38 (25, 51)	52 (13)	68 (62, 74)
1.52 (3.4)	122 (10)	126 (20)	−5 (−11, 2)	119 (12)	2 (−1, 5)	112 (8)	9 (5, 13)	64 (28)	60 (48, 73)	88 (26)	41 (27, 55)	55 (12)	69 (61, 76)
1.61 (3.6)	124 (9)	121 (9)	3 (1, 5)	122 (10)	2 (−1, 4)	115 (8)	10 (7, 12)	69 (27)	58 (45, 70)	89 (24)	43 (31, 56)	59 (11)	68 (62, 74)
1.70 (3.8)	128 (9)	126 (9)	2 (−1, 5)	127 (10)	1 (−1, 4)	118 (7)	10 (6, 15)	75 (28)	56 (41, 70)	97 (26)	41 (25, 56)	59 (12)	71 (65, 77)
1.79 (4.0)	130 (9)	127 (10)	3 (1, 5)	128 (11)	2 (−1, 3)	119 (9)	11 (7, 15)	78 (27)	55 (42, 68)	99 (24)	42 (28, 56)	61 (12)	72 (65, 78)

**Table 2:** Shaker testing frequency, corresponding computed acceleration (g), and mean ± SD steps detected by the Omron HJ720ITC and the Digiwalker SW200 during orbital shaker testing. Total acceleration of 1.21 g is required for the Digiwalker SW200 to detect steps. For the Omron, at a radius of 2.1 cm, steps are not detected till an acceleration of 0.10 g is achieved. Although acceleration is ≥ 0.10 g at radii of 2.6, 4.5, and 12 cm, steps are not detected till a frequency of 1 Hz (60 rpm) is reached.

Device	Radius= 2.1 cm			Radius= 2.6 cm			Radius= 4.5 cm			Radius= 12 cm		
	*Freq. [rpm (Hz)]*	*Steps*	*Acc. (g)*	*Freq. [rpm (Hz)]*	*Steps*	*Acc. (g)*	*Freq. [rpm (Hz)]*	*Steps*	*Acc. (g)*	*Freq. [rpm (Hz)]*	*Steps*	*Acc. (g)*
**Omron**	58 (0.97)	0	0.08	58 (0.97)	0	0.10	58 (0.97)	0	0.17	27 (0.45)	0	0.09
	60 (1.00)	0	0.09	59 (0.98)	0	0.10	59 (0.98)	0	0.18	28 (0.47)	0	0.11
	66 (1.10)	19 ± 3	0.10	60 (1.00)	10 ± 5	0.10	60 (1.00)	31 ± 4	0.18	59 (0.98)	0	0.46
	67 (1.11)	36 ± 6	0.11	61 (1.01)	53 ± 8	0.11	61 (1.01)	61 ± 1	0.19	60 (1.00)	34 ± 4	0.48
	68 (1.13)	67 ± 1	0.11	62 (1.03)	63 ± 1	0.11	62 (1.03)	63 ± 1	0.19	61 (1.01)	61 ± 1	0.50
	70 (1.17)	71 ± 2	0.12	70 (1.17)	70 ± 1	0.14	70 (1.17)	70 ± 1	0.25	70 (1.17)	71 ± 1	0.65
**Digiwalker**	224 (3.73)	0	1.17	201 (3.35)	0	1.17	152 (2.53)	0	1.16	90 (1.50)	0	1.08
	225 (3.75)	0	1.18	202 (3.37)	0	1.18	153 (2.55)	0	1.17	92 (1.53)	0	1.14
	226 (3.77)	0	1.19	203 (3.38)	0	1.19	154 (2.57)	0	1.19	94 (1.57)	0	1.18
	227 (3.78)	23 ± 6	1.21	204 (3.40)	23 ± 5	1.21	155 (2.58)	14 ± 2	1.21	95 (1.58)	20 ± 7	1.21
	228 (3.80)	228 ± 2	1.22	205 (3.42)	204 ± 2	1.22	156 (2.60)	155 ± 4	1.22	96 (1.60)	96 ± 1	1.23
	230 (3.83)	231 ± 4	1.24	207 (3.45)	207 ± 2	1.24	158 (2.63)	157 ± 2	1.25	98 (1.63)	98 ± 1	1.29

**Table 3:** Variable combinations of acceleration frequency and amplitude thresholds necessary to commence step counting using ActiLife software. ‘x’ indicates step detection, while ‘0’ indicates the absence of step detection.

Freq. (Hz.)	0.06 g	0.07 g	0.08 g	0.09 g	0.1 g	0.11 g	0.12 g	0.13 g	0.14 g	0.15 g	0.16 g	0.17 g	0.18 g
**0.2**	0	0	0	0	0	0	0	0	0	x	x	x	x
**0.4**	0	0	0	x	x	x	x	x	x	x	x	x	x
**0.6**	0	x	x	x	x	x	x	x	x	x	x	x	x
**0.8**	0	x	x	x	x	x	x	x	x	x	x	x	x
**1.0**	0	0	x	x	x	x	x	x	x	x	x	x	x
**1.2**	0	0	x	x	x	x	x	x	x	x	x	x	x
**1.4**	0	0	0	x	x	x	x	x	x	x	x	x	x
**1.6**	0	0	0	0	x	x	x	x	x	x	x	x	x
**1.8**	0	0	0	0	0	x	x	x	x	x	x	x	x
**2.0**	0	0	0	0	0	0	0	x	x	x	x	x	x
**2.2**	0	0	0	0	0	0	0	0	0	x	x	x	x
**2.4**	0	0	0	0	0	0	0	0	0	0	0	x	x

## Supplementary Materials

The following are available online at http://www.mdpi.com/1424-8220/18/4/1206/s1, Table S1: Mean ± SD of the second dominant frequencies (Hz) detected in the acceleration signal during the walking protocol.
