# Supplementary material for: “What Is a Step?” Differences in How a Step Is Detected among Three Popular Activity Monitors That Have Impacted Physical Activity Research
Source: Sensors (Basel). 2018 Apr 15;18(4):1206. doi: 10.3390/s18041206 (PMC5948774; doi:10.3390/s18041206)
Supplement: Supplementary file 1 [file sensors-18-01206-s001.zip › Supplementary material/Clean dataset GT9X wrist/Readme.docx]

These selected data are cleaned data that are representative of steady state treadmill walking detected by the ActiGraph GT9X at the wrist. These may serve as a starting point for experts interested in developing wrist –worn algorithms to detect walking. These data will need to be used in conjunction with data collected from a wide range of populations (e.g., age groups) during over-ground ambulatory activities and several other activities of daily living to yield a widely acceptable algorithm to detect steps to increase generalizability.
