# Supplementary material for: “What Is a Step?” Differences in How a Step Is Detected among Three Popular Activity Monitors That Have Impacted Physical Activity Research
Source: Sensors (Basel). 2018 Apr 15;18(4):1206. doi: 10.3390/s18041206 (PMC5948774; doi:10.3390/s18041206)
Supplement: Supplementary file 1 [file sensors-18-01206-s001.zip › Supplementary material/Supplementary table 1.pdf]

	0.89 m/s (2mph)	0.98 m/s (2.2mph)	1.07 m/s (2.4mph)	1.16 m/s (2.6mph)	1.25 m/s (2.8mph)	1.34 m/s (3mph)	1.43 (3.2mph)	1.52 m/s (3.4mph)	1.61 m/s (3.6mph)	1.7 m/s (3.8mph)	1.79 m/s (4mph)
**Hip**	2.75 (1.38)	2.48 (1.66)	2.86 (1.94)	2.30 (1.59)	2.27 (1.65)	2.90 (1.38)	3.01 (1.83)	3.0 (1.61)	3.38 (2.1)	3.54 (1.70)	3.57 (1.40)
**Wrist**	1.71 (0.67)	1.56 (0.76)	1.23 (0.41)	1.24 (0.47)	1.45 (0.85)	1.63 (1.09)	1.37 (0.88)	1.38 (0.89)	1.50 (0.88)	1.41 (0.65)	1.68 (1.06)

Supplementary table 1. Mean ± SD of the second dominant frequencies (Hz) detected in the acceleration signal during the walking protocol.
